# Contrast-enhanced harmonic endoscopic ultrasound–guided pancreatic duct drainage: a practical troubleshooting for the obscured main pancreatic duct

**DOI:** 10.1016/j.vgie.2026.04.054

**Published:** 2026-05-06

**Authors:** Soma Fukuda, Masato Endo, Yusuke Niisato, Yuya Hagiwara, Naoki Ishikawa, Takashi Ishikawa, Taku Sakamoto, Kiichiro Tsuchiya

**Affiliations:** 1 Department of Gastroenterology, Institute of Medicine, University of Tsukuba, Ibaraki, Japan; 2 Graduate School of Comprehensive Human Sciences, University of Tsukuba, Ibaraki, Japan

## Abstract

**Background and Aims:**

EUS-guided pancreatic duct drainage (EUS-PDD) is a vital salvage technique, but identifying the main pancreatic duct (MPD) on B-mode EUS is challenging when it contains isoechoic debris. We aimed to demonstrate a “dual-imaging strategy” to enhance the safety and precision of EUS-PDD for an obscured MPD.

**Methods:**

A 70-year-old woman with pancreatic head cancer presented with obstructive pancreatitis. Transpapillary drainage failed due to complete obstruction. On B-mode EUS, the MPD in the pancreatic tail—the intended puncture site—was entirely obscured by internal debris, making it impossible to identify as a distinct target. To overcome this, we performed contrast-enhanced harmonic EUS (CH-EUS). Simultaneously, color Doppler was used to map peripancreatic vessels.

**Results:**

CH-EUS clearly delineated the MPD as an avascular structure, providing a definitive “black-and-white” roadmap. With real-time CH-EUS guidance, the obscured MPD was precisely punctured with a 22-gauge needle while ensuring a safe tract via color Doppler. After tract dilation, a 6F plastic stent was successfully deployed. The patient's symptoms improved rapidly without adverse events.

**Conclusions:**

Combining CH-EUS for ductal visualization with color Doppler for vascular mapping is a practical strategy to enhance the feasibility of challenging EUS-PDD when the MPD is obscured on conventional B-mode imaging.

EUS-guided pancreatic duct drainage (EUS-PDD) is a salvage technique for obstructive pancreatitis when transpapillary access fails.^1^^,^^2^ However, EUS-PDD remains a technically demanding procedure due to small target sizes, making clear visualization of the main pancreatic duct (MPD) essential for success. In chronic or severe pancreatitis, isoechoic debris can obscure the MPD on B-mode, increasing the risk of mispuncture. We present a case of successful contrast-enhanced harmonic EUS-PDD for an obscured MPD. This study was approved by our Institutional Review Board (no. R07-304).

A 70-year-old woman with pancreatic head cancer undergoing chemotherapy presented with acute abdominal pain and fever. Laboratory data showed an elevated serum lipase level and inflammatory markers. CT revealed a pancreatic head mass causing distal MPD dilatation and parenchymal edematous changes, consistent with acute obstructive pancreatitis (Fig. 1). Given that the pancreatitis precluded chemotherapy, a multidisciplinary team decided that urgent drainage was necessary. Despite the removal of a previously placed biliary fully covered self-expandable metal stent, transpapillary pancreatic duct drainage failed due to complete ductal obstruction by the tumor (Fig. 2).

**Figure 1:**
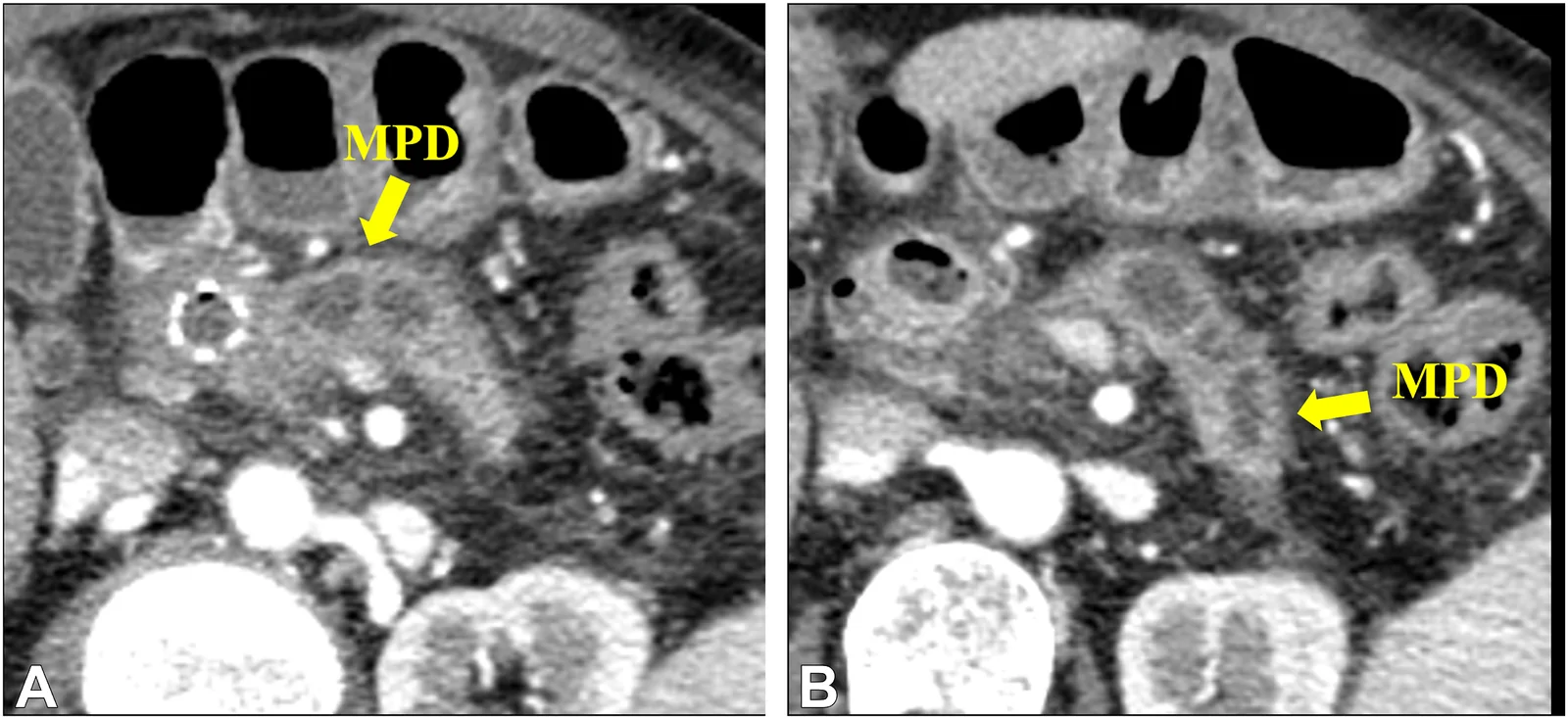
Contrast-enhanced CT images. **A,** Pancreatic body: tumor with distal main pancreatic duct (MPD) dilatation and parenchymal edematous changes. **B,** Pancreatic tail: persistent MPD dilatation with similar edematous changes.

**Figure 2:**
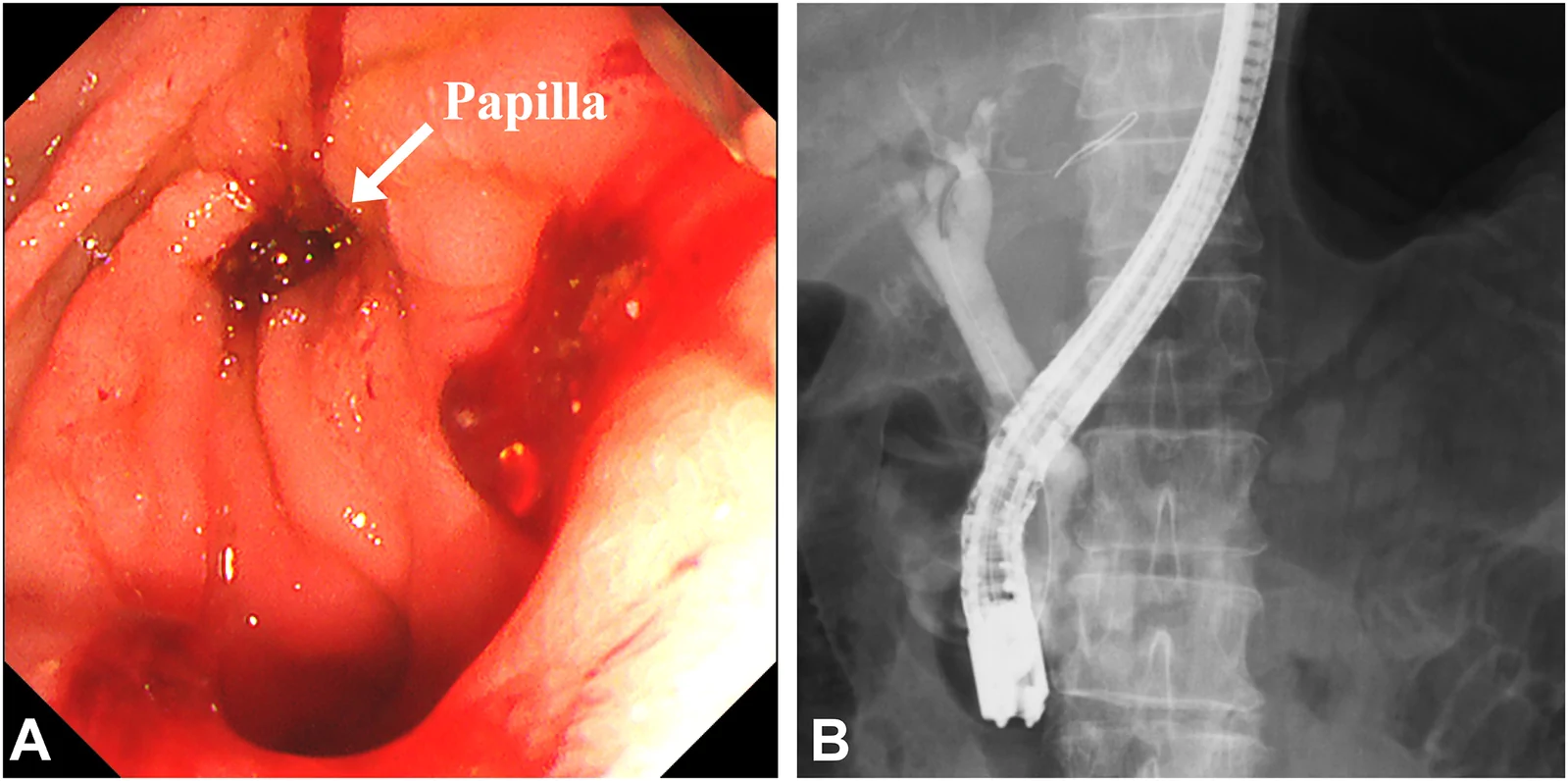
Failed transpapillary access. **A,** Endoscopic view showing no identifiable pancreatic duct orifice after biliary stent removal. **B,** Fluoroscopic image showing contrast in the bile duct but none in the pancreatic duct.

We proceeded with EUS-PDD via a transgastric approach with a linear echoendoscope (GF-UCT260; Olympus, Tokyo, Japan) (Video 1, available online at www.videogie.org). Although the MPD was partially visible on B-mode, isoechoic debris in the tail obscured the ductal margins, precluding safe targeting at the intended site (Fig. 3). To overcome this, we performed CH-EUS on the EU-ME3 (Olympus; mechanical index, 0.28). After confirming the absence of egg allergy in the patient, we administered an intravenous 0.015 mL/kg bolus of perflubutane (Sonazoid; Daiichi Sankyo, Tokyo, Japan). With the resonance of microbubbles, this technique enhanced the parenchyma to delineate the MPD as an avascular “black-and-white” roadmap for approximately 60 seconds, starting 10 to 20 seconds postinjection (Fig. 4). Before puncture, color Doppler mode was used to map peripancreatic vessels, as CH-EUS enhances both parenchyma and vasculature.

**Figure 3:**
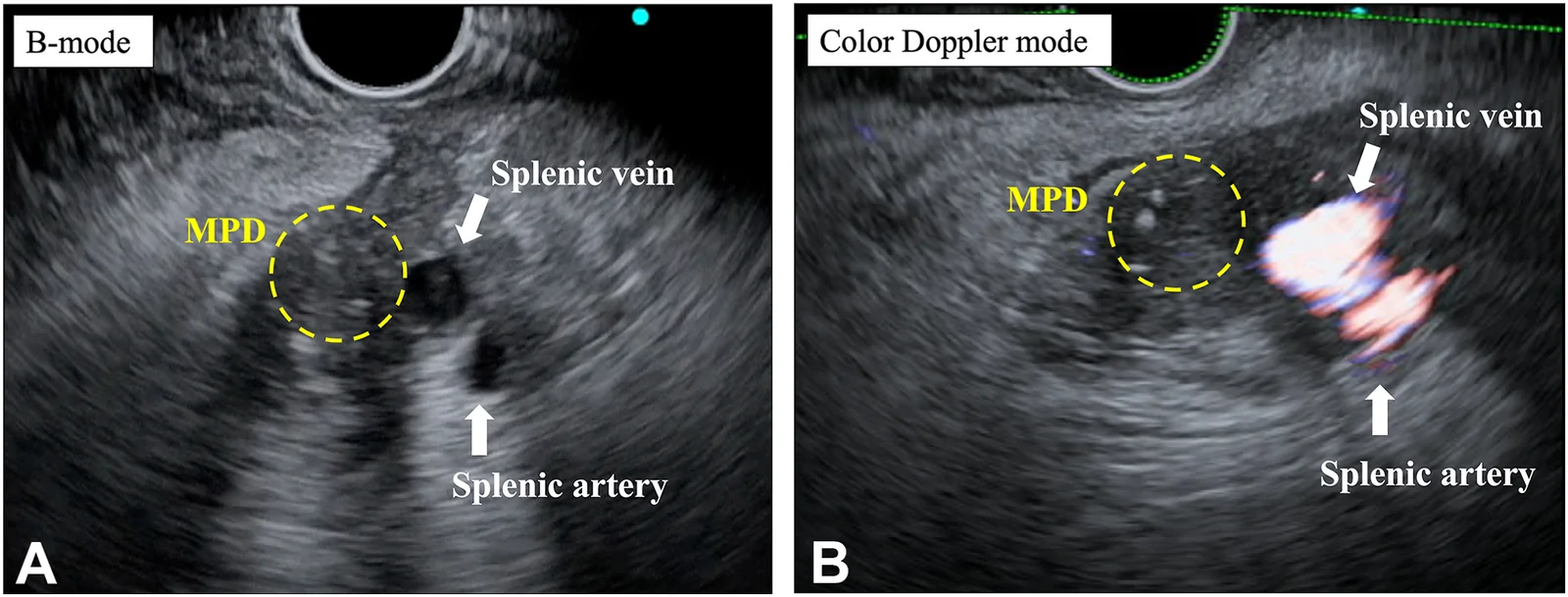
EUS images of the pancreatic tail. **A,** B-mode image shows an isoechoic main pancreatic duct (MPD) with indistinct borders. **B,** Color Doppler mode identifies peripancreatic vessels, while the MPD remains obscured.

**Figure 4:**
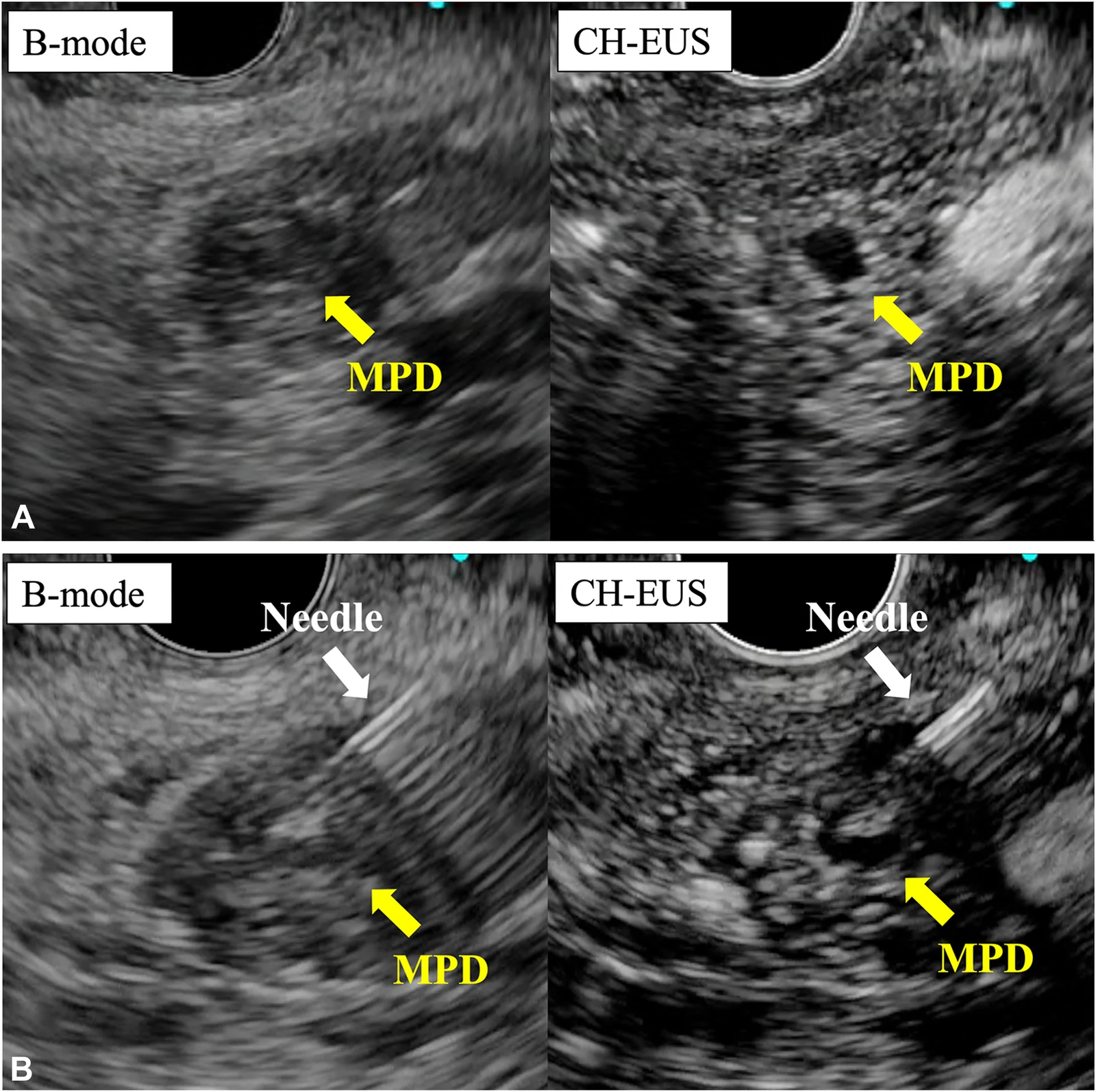
Main pancreatic duct (MPD) puncture under contrast-enhanced harmonic EUS (CH-EUS) guidance. **A,** Although the MPD is poorly visualized on B-mode, CH-EUS clearly delineates the 3.2-mm-diameter duct as an avascular structure. **B,** Real-time visualization of the 22-gauge needle accurately entering the obscured MPD under CH-EUS guidance.

With real-time CH-EUS guidance, the MPD—revealed to be 3.2 mm in diameter—was precisely punctured with a 22-gauge needle (SonoTip ProControl; Medi-Globe GmbH, Rohrdorf, Germany). After contrast confirmation and 0.018-inch guidewire (Fielder 18; Asahi Intecc, Aichi, Japan) insertion, the tract was dilated with a drill dilator (Tornus ES; Asahi Intecc) (Fig. 5). Subsequently, an Uneven Double Lumen Cannula (Piolax Medical Devices, Kanagawa, Japan) was inserted to facilitate the placement of an additional 0.025-inch guidewire (Fielder 25; Asahi Intecc). Although crossing the MPD stricture was difficult, we successfully deployed a 6F, 12-cm plastic stent designed for EUS-PDD (Harmo Ray USR; Silux, Saitama, Japan) from the MPD to the stomach (Fig. 6). Postprocedure CT confirmed appropriate stent placement and resolved MPD dilatation (Fig. 7). No adverse events occurred; rapid symptomatic improvement enabled the resumption of chemotherapy. During 6-month follow-up, no recurrence of obstructive pancreatitis was observed.

**Figure 5:**
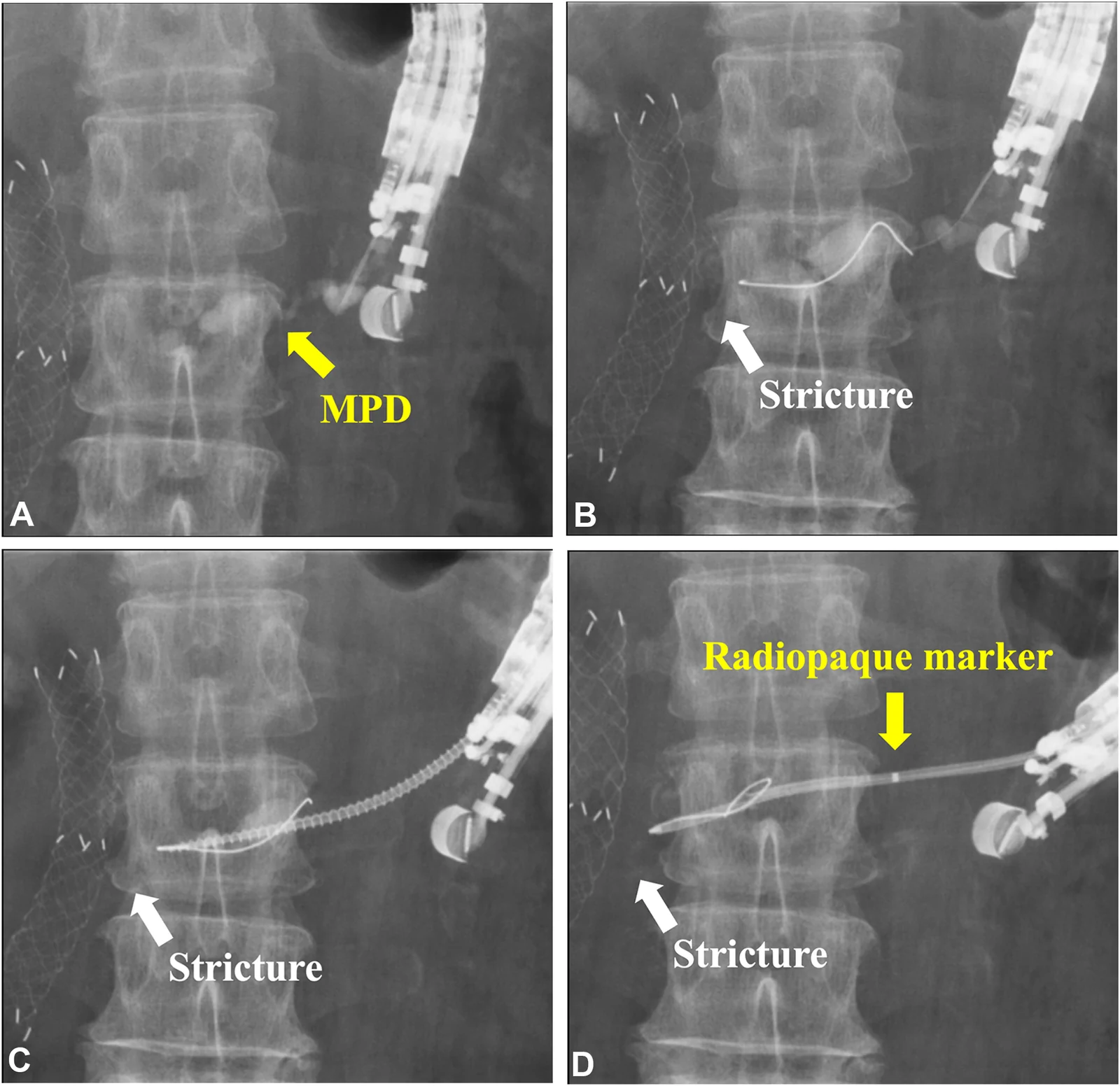
Fluoroscopic images of EUS-guided pancreatic duct drainage (EUS-PDD). **A,** Contrast-filled main pancreatic duct (MPD) after puncture. **B,** Guidewire insertion into the MPD. **C,** Tract dilation using a drill dilator. **D,** Insertion of a 6F plastic stent. The radiopaque marker (one-third from the tip) and distal side holes are positioned within the MPD to optimize drainage and prevent peritoneal leakage.

**Figure 6:**
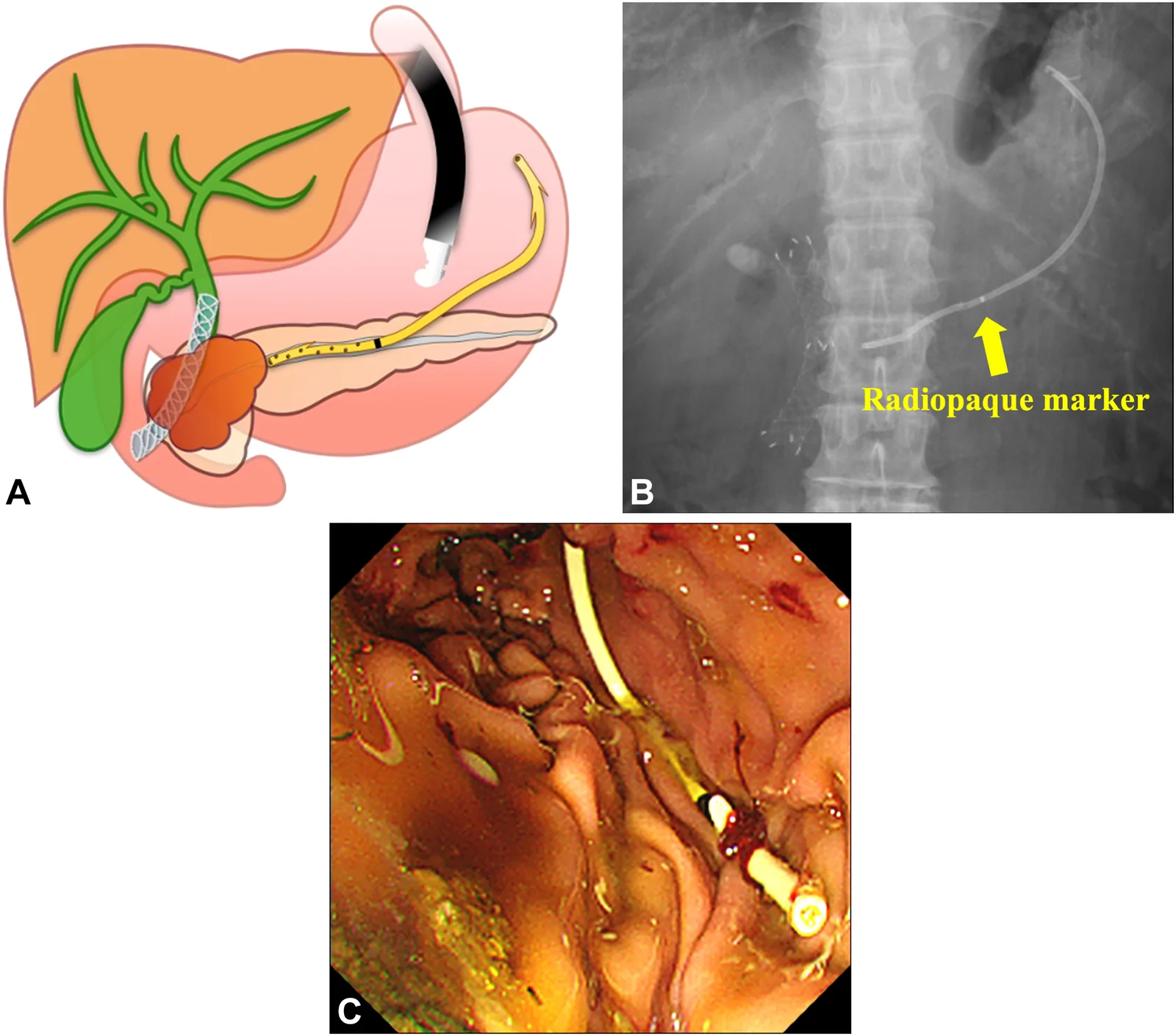
Final configuration of EUS-guided pancreatic duct drainage. **A,** Schematic illustration showing the stent deployment layout. **B,** Final fluoroscopic image showing the successfully placed plastic stent. **C,** Endoscopic view of the stent deployed into the stomach through the gastric wall.

**Figure 7:**
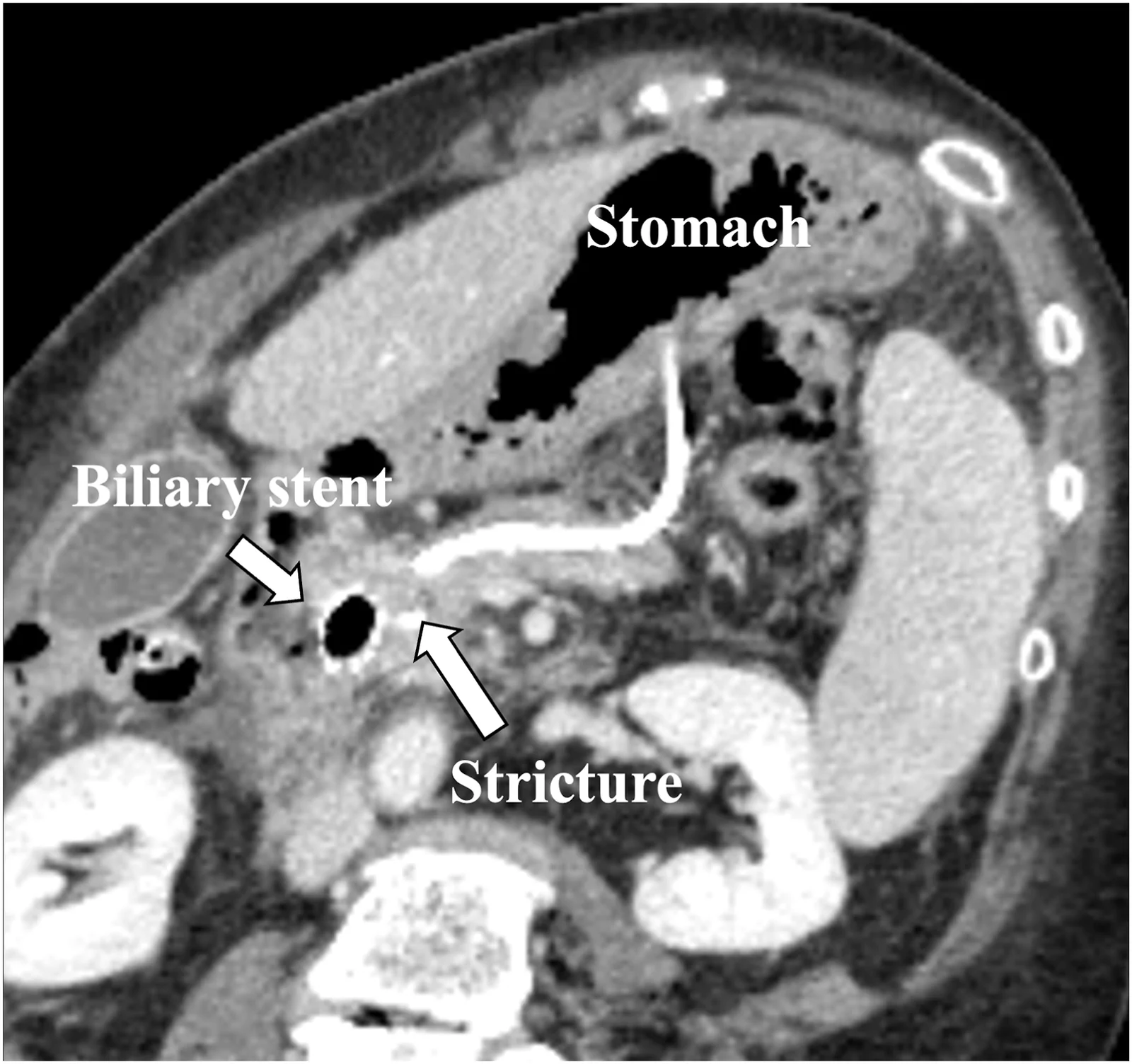
Contrast-enhanced CT after EUS-guided pancreatic duct drainage. Multiplanar reconstruction image showing a plastic stent placed from the stomach to the main pancreatic duct, with resolved ductal dilatation.

Although CH-EUS is well established for characterizing pancreatic masses,^3^, ^4^, ^5^ its role in interventional EUS has expanded to guiding the drainage of fluid collections and the biliary tract.^6^, ^7^, ^8^ Building on these applications, our approach represents a practical technical refinement of EUS-PDD. Unlike larger fluid collections, the MPD is a small target that allows no room for error. A single failed puncture attempt can cause ductal collapse, making further access impossible. Therefore, clear and stable visualization is indispensable. When internal debris makes the MPD indistinguishable from the parenchyma on B-mode, CH-EUS provides the necessary contrast to precisely stabilize the needle tip within the narrow lumen.

Compared with percutaneous pancreatic duct drainage, which involves risks of pancreatic juice leakage and necessitates external drainage,^9^ our refined EUS-PDD ensures safer internal drainage and a better quality of life. However, CH-EUS cannot replace color Doppler for vascular identification because vessels also enhance after contrast injection. Therefore, a dual-imaging strategy—color Doppler for vascular mapping and CH-EUS for ductal visualization—is essential to enhance the safety and feasibility of challenging EUS-PDD.

## Disclosure

All authors disclosed no financial relationships.

## Supplementary data

Video 1 A case of EUS-guided pancreatic duct drainage successfully performed using contrast-enhanced harmonic imaging for an obscured main pancreatic duct.
